# Therapeutic potential of traditional Chinese medicine on heat stroke

**DOI:** 10.3389/fphar.2023.1228943

**Published:** 2023-09-25

**Authors:** Lei Li, Man Wang, Jikuai Chen, Juelin Chen, Yawei Wang, Minghao Zhao, Qing Song, Shuogui Xu

**Affiliations:** ^1^ Department of Emergency, Changhai Hospital, Naval Medical University, Shanghai, China; ^2^ Department of Emergency, The Second Naval Hospital of Southern Theater Command of PLA, Sanya, China; ^3^ Heatstroke Treatment and Research Center of PLA, Sanya, China; ^4^ School of Traditional Chinese Medicine, Naval Medical University, Shanghai, China; ^5^ Department of Health Toxicology, Faculty of Naval Medicine, Naval Medical University, Shanghai, China.; ^6^ Department of Orthopedics Trauma, Changhai Hospital, Naval Medical University, Shanghai, China.; ^7^ Company 11, Regiment 4, College of Basic Medical Sciences, Naval Medical University, Shanghai, China.; ^8^ Department of Critical Care Medicine, First Medical Center, Chinese PLA General Hospital, Beijing, China; ^9^ Department of Critical Care Medicine, Hainan Hospital, Chinese PLA General Hospital, Sanya, China

**Keywords:** heat stroke, traditional Chinese medicine, Chinese patent medicine, alternative therapy, herbal therapy

## Abstract

As global warming progresses, heat waves are becoming increasingly frequent and intense, meanwhile the incidence of heat stroke (HS) has increased sharply during the past decades. HS is typically associated with significant morbidity and mortality, and there is an urgent need for further research to solve this difficult issue. There currently exists difficulties regarding on-site emergency treatment methods and limited in-hospital treatment approaches, and better treatments are required as soon as possible. Theories and therapies from various traditional Chinese medicine (TCM) academic groups have been widely reported. Therefore, an exploration of prevention and protection methods should consider TCM experiences as an alternative. This article primarily reviews TCM herbal therapies and external therapies that have been described in various clinical reports and demonstrated in relevant studies. Herbal therapies, including herbal formulas, Chinese patent medicines (CPMs), single Chinese herbs, and associated extracts or monomers, are summarized based on the shared perspectives of the underlying mechanisms from TCM. In addition, external therapies including acupuncture, bloodletting, cupping, Gua sha and Tui na that have rarely been rarely mentioned and considered in most cases, are introduced and discussed to offer a unique perspective in the search for novel interventions for HS. In summary, TCM may provide abundant potential clinical benefits and research directions in the fight against HS.

## 1 Introduction

Heat stroke (HS) is characterized by severe hyperthermia (typically>40.5 °C), central nervous system dysfunction, and multiple organ failure. According to its cause and susceptible population, HS can be categorized as either classic heat stroke (CHS) or exertional heat stroke (EHS) (Epstein and Yanovich, 2019). Due to uncontrolled global warming, heatwaves, defined as prolonged periods of extreme hot weather or a spell of three or more consecutive hot days, have been proven to be more intense, more frequent and of longer duration. In addition, an extreme hot climate emergency is too severe for human tolerance and has direct immediate effects on human health (Ahima, 2020; Raymond et al., 2020). Therefore, the incidence and mortality of HS reported at various times and locations are ever-increasing. As is well-known, the cornerstone of HS treatment is to control the severe hyperthermia by performing rapid and effective cooling that can significantly alleviate the deterioration of disease. However, in most cases, on-site emergency treatment cannot access cooling equipment in the open, which indicates that HS patients with uncontrollable hyperthermia would soon develop dreadful systematic inflammatory response system (SIRS) that leads to multiple organ failure and death (Liu et al., 2020). Furthermore, few pharmacologic agents have been demonstrated to be able to accelerate cooling. Patients with confirmed or suspected HS should be transported to the nearest hospital as soon as possible to receive advanced in-hospital treatment. Although patients can obtain intensive care and various treatments for organ injuries in hospital, the prognosis for the majority tends to be rather poor without any specific therapy. In addition, many of them will suffer a heavy medical burden due to multiple treatment. Given the limited therapies and poor prognosis of HS, additional therapies are required to improve the therapeutic effect by focusing on alternative or complementary therapies (Li et al., 2021).

Traditional Chinese medicine (TCM), as an empirical medicine based on the unique theoretical system of TCM, has a long history and clinical practice on HS(Li et al., 2022a). Additionally, numerous records in the ancient TCM literature have been found to provide potential detailed beneficial therapies in detail for reference in detail (Li and Liang, 2017). Under the guidance of TCM theory, the therapeutic methods on HS can be classified into drug therapies and external therapies according to whether using traditional Chinese herbal medicine. Herbal therapies, including herbal formulas, Chinese patent medicines (CPMs), single Chinese herbs, and associated extracts or monomers have been applied and researched to treat HS throughout Chinese history (Qiu et al., 2020). In addition, external therapies including acupuncture, bloodletting, cupping, Gua sha and Tui na that have been rarely mentioned and considered in most cases, have been reported to have unique relief effects against HS. With the development of the integration of TCM and Western medicine, an increasing number of herbs have been investigated using rigorous experiments. The results have indicated, that these herbs or herbal drugs that have been proven to be effective can be developed and promoted to help clinicians fight against HS. To thoroughly review the role of TCM in the treatment of HS, the following electronic databases will be searched from the respective dates of database inception to 30 April2023: the Cochrane Library, PubMed, the Web of Science, the EMBASE, the China Science and Technology Journal, the China National Knowledge Infrastructure, the Wanfang, the Chinese Biomedical Literature Database, and other sources. Given that much of the literature on TCM treatment for HS was first reported in Chinese journals, we first analyzed the Chinese literature and then conducted a comparative analysis combined with ancient TCM books. Subsequently, a literature search was conducted in the English databases. Finally, all literature related to the treatment to HS with TCM were classified, discussed, and reviewed.

In this review, we introduce the ancient records and basic theory of TCM on HS to provide a new look at this life-threatening disease. In addition, we summarize the drug therapies and external therapies of TCM to provide a number of alternative or complementary therapies with therapeutic potential on HS for the prevention and protection. Finally, the relevant herbal therapies are classified and analyzed to determine the key herbal drugs that have the significant research value. This review provides a new understanding of HS in the view of TCM and elucidates a series of effective therapies to prevent the onset of HS and alleviate the critical state of patients.

## 2 Ancient records and basic theory of TCM on HS

According to global medical history records, HS, also is known as Sun stroke or heat apoplexy, was first described by a Roman in 24 BC. during an expedition into Arabia Felix (Bouchama, 1995; Casa et al., 2010). Owing to the long history of TCM and the Four Great Inventions in ancient China, relevant records on HS date back to more than 5,000 B.C. in China, and it was not until modern times when Western medicine was introduced into China that there appeared a precise concept of HS. Hence, sorting out and summarizing potential therapies of TCM to have a new look at this ancient disease may help clinicians find solutions on HS from an integrative perspective of TCM and Western medicine.

*Huangdi Neijing* (*Inner Canon of the Yellow Emperor* or *Esoteric Scripture of the Yellow Emperor*), as the oldest classic TCM text of internal medicine written around 2600 BC, first described this disease caused by heat stress and even classified the various phenotypes of HS according to the pathogenesis (Ye and Dong, 2017). Since then, countless medical theories and therapies of HS have been recorded in plenty of classic TCM texts (Li and Liang, 2017; Capodice and Chubak, 2021; Si and Si, 2022), such as *Wenbing Lun* (*Treatise on Warm-heat Diseases*), *Jingui Yaolue* (*Synopsis of Prescriptions of the Golden Chamber*), and *Wenbing Tiaobian* (*Detailed Analysis of Epidemic Warm Diseases*). In summary, ancient doctors of TCM realized that people’s heat tolerance is limited and once a person in a hot environment suffers from too much heat stress, they can easily develop HS. Therefore, the theory indicated that heat stress is a type of external injury, and HS patients require the administration of heat-clearing herbal drugs or the adoption of external therapies to release heat from the body. This became the consensus in TCM. In addition, ancient doctors of TCM found that patients at the end stage of HS urgently required medical support as much as possible, and they tried to use various herbal drugs to strengthen the body resistance. Interestingly, modern Western medicine recommends that hospitalized patients with HS should be cared for in intensive care unit (ICU) to obtain systematic supportive therapy to treat SIRS and multiple organ injuries (Liu et al., 2020). Guided by the theory above, lots of TCM physicians accumulated various types of herbal formulas through a millennial of experience, and this saved numerous HS patients in ancient China. Therapeutic methods used in TCM are primarily composed of drug therapies and external therapies. According to the classic theory of TCM that the same disease should be treated with different therapies based on the patients’ actual condition, doctors of TCM generally adopt the comprehensive therapy. In the past, doctors of TCM tended to use herbal formulas in practice, as the combination of several types of medicinal herbs or minerals could strengthen the therapeutic effects, achieve multiple therapeutic targets, and minimize the potential adverse effects. Today, to realize the standardization of TCM drugs, herbal formulas in TCM are modernized into a ready-to-use form such as pills, tables, oral solutions, or injections, which are comprised of multiple herbal extracts and thus are referred to as CPMs. In addition, several types of CPMs have been proven to be effective on HS according to many clinical reports, and these can be easily administrated and transported. Due to the complex ingredients of herbal formulas and their associated mechanisms against HS, an increasing number of studies are currently focusing on the single Chinese herbs and their extracts or monomers from those certified herbal formulas using scientific pharmacological analysis methods. Hence, many single Chinese herbs have been reported to be effective in many articles. In addition, their identified extracts or monomers are easier to accept and promote due to scientific experiments rather than based on ancient experiences. Finally, external therapies, such as Gua sha, Tui na, and acupuncture, have been deemed to be able to purge internal heat and cool or refresh body with unexpected beneficial effects on some HS patients.

## 3 Herbal formulas of TCM against HS

Herbal therapy of TCM can be characterized using various multi-herb formulas that were widely applied in ancient China. Herbal formulas are a combination of herbs used in TCM herbology to obtain more efficiency compared with single Chinese herbs. In fact, numerous herbal formulas were discovered in ancient classics of TCM to treat HS. Through long-term clinical practice, several herbal formulas became golden therapeutic options and were passed down to now. Hence, six classical herbal formulas that have been demonstrated in published scientific articles are introduced and discussed in this manuscript.

### 3.1 Huoxiang Zhengqi San

Huoxiang Zhengqi San (HXZQS) is a classic formula recorded in the *Taiping Huimin Hejiju Fang* (*Prescriptions of Peaceful Benevolent Dispensary*) that is composed of 10 main herbs with information all listed in Table 1 (Xiao et al., 2020). HXZQS has been known for more than 900 years to have the effects of inducing diaphoresis, clearing heat, resolving damp, and regulating the functions of the spleen and stomach. This formula has various dosage forms, such as capsules, granules, oral liquid, and pills (Li et al., 2022b). As all these dosage forms work through the same formula of HXZQS, and they should be classified as TCM formula prescriptions, and cannot be classified as CPMs. A study found that HXZQS could significantly protect intestinal barrier function and prevented acute intestinal injury induced by HS(Li et al., 2022a). This study also found that HXZQS could perform these effects by increasing the expression of claudin-3 that could strengthen the intestinal barrier. HXZQS was also recommended as a hyperthermia relief medicine to provide post-disaster medical rescue strategy in tropical regions (Fan et al., 2012). In fact, a clinical study demonstrated that HXZQS could significantly decrease the level of endotoxin and increase the concentration of heat shock proteins in 84 patients with HS(Wang et al., 2014a). Another clinical study found that taking HXZQS before training could remarkably lower the incidence of HS(Li et al., 2016). In addition to these studies directly demonstrating protective effects against HS, accumulated evidence has shown that HXZQS could ameliorate various injuries induced by hyperthermia in many febrile diseases. A randomized controlled trial also found that HXZQS had advantages for COVID-19 patients in improving clinical symptoms and reducing the dosage of anti-infective drugs (Xiao et al., 2020). In addition, many studies of HXZQS have demonstrated its potent gastrointestinal protective effects (Dong et al., 2022), and gastrointestinal injury has been shown to play a key role in the pathophysiological development of HS.

**TABLE 1:** Summary of representative herbal formulas for HS.

Herbal formulas	Main compositions		Therapeutic effects	References
Huoxiang Zhengqi San	Baizhi	*Angelica dahurica (Hoffm.) Benth. & Hook.f. ex Franch. & Sav.*	Induce diaphoresis, clear heat, resolve damp and regulate the function of the spleen and stomach	Fan et al. (2012), Li et al. (2022a)
(HXZQS)	Banxia	*Pinellia ternata (Thunb.) Makino*		
	Cangzhu	*Atractylodes lancea (Thunb.) DC.*		
	Chenpi	*Citrus reticulata Blanco*		
	Dafu	*Areca catechu L.*		
	Fuling	*Poria cocos(Schw.)Wolf*		
	Gancao	*Glycyrrhiza uralensis Fisch. ex DC.*		
	Houpu	*Houpoea officinalis (Rehder & E.H.Wilson) N.H.Xia & C.Y.Wu*		
	Huoxiang	*Agastache rugosa (Fisch. & C.A.Mey.) Kuntze*		
	Zisu	*Perilla frutescens (L.) Britton*		
Shenmai San	Maimendong	*Ophiopogon japonicus (Thunb.) Ker Gawl.*	Treatment of Qi and Yin deficiency	Zhang et al. (2022b)
(SMS)	Renshen	*Panax ginseng C.A.Mey.*		
	Wuweizi	*Schisandra chinensis (Turcz.) Baill.*		
Xingjun San	Bingpian	*Borneol*	Induce resuscitation, clear heat, repel foulness with aromatics and remove toxicity	Zhang (2018a); Zhang (2018b), Yang (2019)
(XJS)	Niuhuang	*Calculus bovis*		
	Pengsha	*Borax*		
	Shengjiang	*Zingiber officinale Roscoe*		
	Shexiang	*Deer musk*		
	Xiaoshi	*Niter*		
	Xionghuang	*Realgar*		
	Zhenzhu	*Pearl*		
Xiangru San	Baibiandou	*Lablab purpureus (L.) Sweet*	Dispel summer-heat, relieve exterior syndrome, resolve damp and harmonize the spleen and stomach	Huang, 2010; Zhu et al. (2020)
(XRS)	Houpu	*Houpoea officinalis (Rehder & E.H.Wilson) N.H.Xia & C.Y.Wu*		
	Xiangru	*Elsholtzia ciliata (Thunb.) Hyl.*		
Baihu Decoction	Gancao	*Glycyrrhiza uralensis Fisch. ex DC.*	Remove heat and promote the production of body fluids	Wang et al. (2020a), Ping et al. (2020)
(BHD)	Jingmi	*Oryza sativa L.*		
	Shigao	*Gypsum*		
	Zhimu	*Anemarrhena asphodeloides Bunge*		
Qingshu Yiqi Decoction	Xiyangshen	*Panax quinquefolius L.*	Remove summer-heat, reinforce Qi, nourish Yin, promote the production of body fluids	Yang (2015)
(QSYQD)	Shihu	*Dendrobium nobile Lindl.*		
	Maimendong	*Ophiopogon japonicus (Thunb.) Ker Gawl.*		
	Huanglian	*Coptis chinensis Franch.*		
	Zhuye	*Phyllostachys nigra (Lodd. ex Lindl.) Munro*		
	Hegeng	*Nymphaea tetragona Georgi*		
	Zhimu	*Anemarrhena asphodeloides Bunge*		
	Gancao	*Glycyrrhiza uralensis Fisch. ex DC.*		
	Jingmi	*Oryza sativa L.*		
	Xiguacuiyi	*Citrullus lanatus (Thunb.) Matsum. & Nakai*		

### 3.2 Shenmai San

Shenmai San (SMS) is a classic formula that consists of three herbs first recorded in the *Yixue Qiyuan* (*Source Principles of Medicine*) in the 12th century (Zhang et al., 2022a) that covers a variety of situations involving fluid and energy loss (Table 1). In an echo of modern western medicine, ancient TCM doctors believed that a loss of fluid and Qi (typically translated as “vital energy”) was responsible for the for the development of an unconsciousness of critical HS patients. In addition, modern medical investigations have clearly proven that the loss of fluid and redistribution of blood flow of visceral organs induced splanchnic ischemia and hypoxia injury (Leong and Ko, 2018). Additionally, precise rehydration therapy and multiple organ support therapy are extremely necessary to save HS patients. A study based on HS rat model found that SMS could alleviate heat stress-induced liver injury via regulating energy metabolism and the AMPK/Drp1-dependent autophagy process (Zhang et al., 2022b). This study also demonstrated that SMS could effectively regulate glycolysis and tricarboxylic acid cycle to relieve energy metabolism disorder. Several early studies have firmly demonstrated that SMS could treat nitric oxidative stress and ischemic injuries in HS rats (Wang et al., 2005a; Wang et al., 2005b; Lee et al., 2005; Chang et al., 2007). These studies found that pretreatment with SMS could significantly attenuated increased plasma levels of iNOS-dependent NO(Wang et al., 2005a). Moreover, SMS was proven to enhance the effect of ginsenosides in upregulating the glucocorticoid receptor in rats (Lu et al., 2009). In fact, SMS as an herbal formula used for the treatment of fluid and Qi deficiency has long been recommended as a key therapy against the depletion of Qi and body fluids in the presence of HS.

### 3.3 Xingjun San

Xingjun San (XJS), also known as troop-marching powder, originated from the team of the Shu State in The Three Kingdoms period and was invented by Zhuge Liang (Zhang, 2018a). Due to the hot and humid environment of jungle, massive military soldiers developed HS and some other heat-related illnesses such as diarrhea, intoxication, and rash. Thanks to the application of XJS, the army was protected from HS and won the battle. Since XJS has the effects of inducing resuscitation, clearing heat, repelling foulness with aromatics and removing toxicity, XJS was further verified to possess protective effects based on modern medical experiments. Previous studies have found that XJS has protective effects against HS-induced liver, stomach, and intestinal injuries by down-regulating the expression of inflammatory factors and up-regulating the levels of HSP70 and the barrier protein ZO-1 (Zhang, 2018a; Zhang, 2018b; Yang, 2019). However, the mechanism of XJS has not been detected, and the entire protective and preventive effects are worth further exploration.

### 3.4 Xiangru San

Xiangru San (XRS) is another classic formula recorded in the *Taiping Huimin Hejiju Fang* (*Prescriptions of Peaceful Benevolent Dispensary*), and it consists of the three herbs listed in Table 1 (Han et al., 2017; Li et al., 2020a). XRS also has the effects of dispelling summer-heat, relieving exterior syndrome, resolving dampness, and harmonizing the spleen and stomach. Researchers have found that the use of thermal moxibustion combined with XRS could significantly improve the symptom scores of mental exhaustion, dizziness, chest tightness, fever, nausea, vomiting and so on of HS patients (Zhu et al., 2020). In addition, XRS was usually made in ancient times as a drink with a sweet taste that helped people resist the hot climate and fight against HS. From 2007 to 2008, 70 cases of HS children with hyperthermia were treated with XRS, and satisfactory results were obtained that included a faster cooling rate and strength rehabilitation (Huang, 2010).

### 3.5 Baihu Decoction

Baihu Decoction (BHD) is a type of herbal decoction that is used as an antipyretic medicine with the famous effects of removing heat and promoting the production of body fluids and was first recorded in the *Shanghan Lun* (*Treatise on Cold Damage Diseases*) (Wang et al., 2022a). BHD, also known as White Tiger Decoction, has been extensively used in the early stage of acute infection and inflammation. Therefore, BHD and its modified formula are often used to treat HS patients in China. It has been proven that BHD could inhibit the expression of several inflammatory proteins such as IL-1β, TNF-α and NF-κB which indicated that BHS may exert its effects through anti-inflammation (Ping et al., 2020). Another study found that BHD can effectively alleviate liver tissue damage caused by heat stress by reducing the heat shock protein 70, and it has been shown to alleviate oxidative damage and inflammation in the liver in experimental acute heat stress mice (Wang et al., 2020a). Furthermore, the prescription of BHD was often modified by adding some other herbs such as Renshen (*Ginseng*), Guizhi (*Ramulus Cinnamomi*). BHD and modified BHD formulas have been demonstrated to remodel the gut microbiota and, inhibit TLR4/NFκB/NLRP3 activation-induced pyroptosis to fight against various inflammation-related diseases (Li et al., 2022b).

### 3.6 Qingshu Yiqi Decoction

Qingshu Yiqi Decoction (QSYQD) is a summer-heat clearing formula, which has the effects of clearing summer-heat, reinforcing Qi, nourishing Yin (usually seen as " restorative energy”) and generating fluid (Xia et al., 2021). This herbal remedy contains nine primary components in Table 1 and is usually translated as Decoction for Clearing away Summer-heat and Reinforcing Qi. According to this translation, this decoction is a direct treatment for HS and is recorded in the *Wenre Jingwei* (*Warp and Weft of Warm and Hot Disorders*) (Song and Wang, 2021). A study on the theory of summer heat based on the thought of ‘air flow over time’ in the *Huangdi Neijing* found that QSYQD was able to reduce the adverse effects of a high-temperature environment on exercise capacity and significantly prolong the time to exhaustion (Sun and Han, 2019; Junwei et al., 2021). Furthermore, it was also found to reduce the content of MDA in skeletal muscles of rats, reduce lipid peroxidation, increase heat superoxide dismutase (SOD) activity, and effectively removed free radicals (Yang, 2015). With the development of TCM, QSYQD can be subdivided into two distinct formula prescriptions that include Wang’s QSYQD and Li’s QSYQD. Wang’s formula concentrates on the treatment of spleen and stomach by improve gastrointestinal function of HS patients. While Wang’s formula focused on the deficiency of Qi and Yin and could effectively supplement the body fluid of HS patients. Studies have shown that QSYQD could effectively resist endotoxemia and improve the prognosis of HS patients (Zhu, 2006; Wang and Liu, 2014).

## 4 Chinese patent medicines of TCM against HS

As mentioned above, the Chinese patent medicines system is based on classical herbal formulas that could be modernized into a ready-to-use forms such as pills, tables, oral solutions, or injections (Yang et al., 2021). Various CPMs have been reported to be protective and preventive against HS. These CPMs can be mainly divided into two categories depending on whether they are administered orally. The CPMs not used orally are usually injections extracted from herbal formulas. The oral CPMs include capsules, granules, solutions, and pills. CPMs prepared using modern technology based on traditional herbal formulas can expand the indication, standardize the ingredients, and lower the threshold for use. However, many CPMs have not been tested through the rigorous clinical trials, and some adverse reactions have occurred, inducing criticisms. Generally, the application of CPMs could help alleviate the injury of HS and improve prognosis according to the available research. Therefore, the CPMs against HS will be introduced according to the two application modes and discussed at the end of this article.

### 4.1 Injections of Chinese patent medicines

As shown in Table 2, four types of CPM injections have been reported to be able to treat HS. They are the Fufangshexiang Injection (FFSXI), the Shenmai Injection (SMI), the Tanreqing Injection (TRQI), the Xingnaojing Injection (XNJI), and the Xuebijing Injection (XBJI). FFSXI was developed on the basis of the herbal prescription, Angong Niuhuang, and could awaken the mind, calm the mind and relieve heat stroke-induced coma (Liu et al., 2015; Hwang et al., 2021). Early research found that FFSXI could alleviate the disturbance of consciousness of HS patients without adverse reaction (Song, 2011). SMI originated from the classic TCM formula SMS mentioned above and has the similar effects of replenishing Qi-Yin deficiency (Wang et al., 2020b). SMI has also been shown to reduce apoptosis, attenuate autophagy, and improve ischemia and reperfusion injury via modulation of the AMPK, mTOR and JNK pathways (Yang et al., 2016; Liu et al., 2018a). In clinical practice in China, various studies have reported that SMI a significant positive effect on the treatment of HS by replenishing Qi-Yin deficiency (Dai, 1997; Dai et al., 2004; Mei et al., 2006; Zhang, 2014; Zhu, 2014). TRQI is an innovative CPM composed of several herbs with the effects of clearing heat, dissipating phlegm, and detoxifying the body (Hu et al., 2022). According to a study, the use of TRQI in the treatment of HS can enhance the cooling effect and promote the recuperation of the patient’s condition (Jin, 2011). Another study discovered that TRQI exhibited favorable antipyretic and wake-promoting effects, while also mitigating the incidence of complications (Yu et al., 2017). XNJI, another derivative CPM of the herbal prescription Angong Niuhuang, has been extensively employed as an emergency intervention in cases of acute cerebral injury or infarction and stroke (An et al., 2022; Wang et al., 2022b). A study indicated that XNJI has the potential to mitigate cerebral ischemia/reperfusion injury through the inhibition of the inflammatory response mediated by SIRT1(Zhang et al., 2020). The administration of XNJI has demonstrated efficacy in reducing the duration of consciousness disorder and hyperthermia resulting from military training (Hu et al., 2021a). Moreover, the combination of early continuous blood purification and Xingnaojing injection has been found to be a highly effective treatment for severe heat stroke (Wang et al., 2019a). This therapeutic approach rapidly reduces core body temperature, stabilizes blood pressure, ameliorates consciousness disturbances in patients, and enhances pulmonary oxygenation and whole-body perfusion. Finally, XBJI, a CPM injection composed of five herbs, has demonstrated efficacy in treating a range of systemic inflammatory diseases by modulating the inflammatory response, reducing oxidative stress, and enhancing coagulation and the immune function (Qiang et al., 2021). Numerous studies in the literature have demonstrated the efficacy of XBJI in mitigating the risk of HS. According to a study, XBJI was observed to mitigate liver injury in HS rats by impeding the secretory function of Kupffer cells (Chen et al., 2013). XBJI also could attenuate HS-related hypotension by upregulating the angiotensin II receptor-associated protein 1 in rats (Pan et al., 2014). XBJI was shown to enhance the endothelial barrier function via PAR1 signaling against HS-induced vascular injury (Xu, 2015). It also has protective effects through decreasing reactive oxygen species (ROS) levels and modulating the gut microbiota (Chen et al., 2017). In addition, clinical research also firmly proved that XBJI could significantly relieve the symptoms of HS and reduce the degree of systemic inflammatory response in multiple injured organs (Jiang, 2018).

**TABLE 2:** Summary of representative CPMs for HS.

Drug name	Main compositions	Therapeutic effects	References
Fufangshexiang Injection	*Deer musk, Curcuma aromatica Salisb, Pogostemon cablin (Blanco) Benth, Acorus gramineus Aiton, Borneol, Menthol.*	Awake the mind and calm the mind, relieve heat stroke-induced coma	Song (2011)
(FFSXI)			

Shenmai Injection	*Panax ginseng C.A.Mey, Ophiopogon japonicus (Thunb.) Ker Gawl, Schisandra chinensis (Turcz.) Baill.*	Replenish Qi-Yin deficiency	Zhang (2014); Zhu (2014)
(SMI)			
Tanreqing Injection	*Scutellaria baicalensis Georgi, Lonicera japonica Andrews, Forsythia suspensa (Thunb.) Vahl.*	Clear heat, dissipate phlegm and detoxify	Jin (2011)
(TRQI)			

Xingnaojing Injection	*Deer musk, Curcuma aromatica Salisb, Borneol, Gardenia jasminoides J.Ellis.*	Clear heat and detoxify, cool blood, awaken brain	An et al. (2022), Wang et al. (2022a)
(XNJI)			
Xuebijing Injection	*Carthamus tinctorius L., Paeonia lactiflora Pall, Ligusticum striatum DC., Salvia miltiorrhiza Bunge, Angelica sinensis (Oliv.) Diels.*	Regulate inflammatory response and oxidative stress, improve coagulation and immune function	Chen et al. (2013), Pan et al. (2014)
(XBJI)			

Kangshire Capsule	*Panax ginseng C.A.Mey, Gypsum, Anemarrhena asphodeloides Bunge.*	Clear heat, relieve heat, and resolve damp.	Zhao et al. (2006), Xu and Zhang (2007)
(KSRC)			
Tongxinluo Capsule	*Panax ginseng C.A.Mey, Paeonia lactiflora Pall., Santalum album L., Ziziphus jujuba Mill, Borneol.*	Invigorate Qi and activate blood circulation	Turson et al. (2021)
(TXLC)			

Yiqing Capsule	*Coptis chinensis Franch, Scutellaria baicalensis Georgi, Rheum palmatum L.*	Clear heat, remove blood stasis., detoxify.	Song et al. (2003)
(YQC)			
Reduping Capsule	*Gypsum, Lonicera japonica Andrews, Scrophularia ningpoensis Hemsl, Rehmannia glutinosa (Gaertn.) DC, Forsythia suspensa (Thunb.) Vahl, Gentiana scabra Bunge, Isatis tinctoria L, Anemarrhena asphodeloides Bunge, Ophiopogon japonicus (Thunb.) Ker Gawl.*	Clear heat, remove, detoxify	Liang et al. (2008a), Yang et al. (2014a)
(RDPC)			





Shidishui Oral Solution	*Mothball, Zingiber officinale Roscoe, Rheum palmatum L, Foeniculum vulgare Mill, Cinnamomum cassia (L.) J.Presl, Capsicum annuum L., Eucalyptus globulus Labill.*	Clear heat, invigorate the spleen	Shen et al. (1998)
(SDS)			



Angong Niuhuang Pill	*Calculus bovis, Deer musk, Pearl, Cinnabar, Realgar, Coptis chinensis Franch, Scutellaria baicalensis Georgi, Gardenia jasminoides J.Ellis, Curcuma aromatica Salisb, Borneol.*	Remove heat for resuscitation, eliminate phlegm and toxic material	Shen et al. (2020), Shi et al. (2021b)
(AGNHP)			




Longhu Rendan Pill	*Menthol, Borneol, Syringa oblata Lindl., Wurfbainia villosa (Lour.) Škorničk. & A.D.Poulsen, Illicium verum Hook.f, Cinnamomum cassia (L.) J.Presl, Piper nigrum L, Dolomiaea costus (Falc.) Kasana & A.K.Pandey, Zingiber officinale Roscoe, Senegalia catechu (L.f.) P.J.H.Hurter & Mabb, Glycyrrhiza uralensis Fisch. ex DC, Oryza sativa L.*	Clear heat, awaken brain, invigorate the spleen	Li et al. (2009)
(LHRDP)			



Wuzi Yanzong Pill	*Lycium chinense Mill, Cuscuta chinensis Lam, Rubus idaeus L, Schisandra chinensis (Turcz.) Baill, Plantago asiatica L.*	Tonify the kidney and secure essence	Hu et al. (2021a), Xu et al. (2021)
(WZYZP)			



### 4.2 Oral Chinese patent medicines

As traditional herbal formulas have the disadvantages of complex preparation and they cannot be standardized, their toxicity and side effects cannot be determined. The preparation of standardized capsules from traditional herbal formulas can play an original therapeutic role, and also meet the requirements of modern drug use on the other hand. As shown in Table 2, nine types of oral CPMs have been reported to be helpful for HS. They are the Kangshire Capsule (KSRC), the Tongxinluo Capsule (TXLC), the Yiqing Capsule (YQC), the Reduping Capsule (RDCP), the Shidishui Oral Solution (SDS), the Angong Niuhuang Pill (AGNHP), the Longhu Rendan Pill (LHRDP) and the Wuzi Yanzong Pill (WZYZP). KSRC, also translated as the anti-damp and heat capsule, is a type of CPM specially used to antagonize damp and heat and developed by Chinese military medical research institutes (Zhao et al., 2006). It originated from BHD and had the effects of antipyretic, anti-inflammatory, immune regulation. Several local studies have indicated that KSRC could protect HS-induced brain injury and improve the thermoregulatory function in hot environments by increasing the levels of the α-melanocyte-stimulating hormone, β-endorphin and decreasing blood levels of SOD and MDA in blood (Xu, 2002; Zhao and Xu, 2004a; Zhao and Xu, 2004b; Zhao, 2004; Zhao et al., 2006; Xu and Zhang, 2007). TXLC, a compound in the form of a capsule widely used in the treatment of various cardiovascular diseases, has the effects of invigorating Qi and activating blood circulation (Xiong et al., 2022). TXLC exhibited a protective effect on cardiomyocytes and facilitated mitochondrial autophagy while suppressing the inflammatory response of myocardial tissues in HS rats due to the activation of the PINK1/Parkin pathway (Turson et al., 2021). In addition, some clinical research has found that TXLC could alleviate the symptoms of HS and especially protect liver and kidney by decreasing the levels of Cr, BUN, CK and ALT (Sun et al., 2014; Wang and Fu, 2019). YQC is a Chinese herbal medicine compound preparation and that has the functions of heat-clearing and detoxification (Xu et al., 2022). This capsule is appropriate for addressing symptoms such as body heat irritability, ocular redness and soreness, oral discomfort, throat soreness, gingival swelling and pain, and constipation. A Chinese clinical report found that YQC could help HS patients by promoting intestinal excretion of heat (Song et al., 2003). Another Chinese clinic that examined 132 HS patients found that YQC could increase the cooling rate and alleviate the organ tissue injury (Liu, 1999). RDPC is a typical heat-clearing and detoxifying CPM and its name could be understood as the elimination of heat toxicity, which means that RDPC directly combat heat damage to the body (Liang et al., 2008a). RDPC could effectively reduce the apoptosis of macrophages and the level of free Ca^2+^concentration under a hot environment, which help treat HS(Yang et al., 2014a). According to another study, RDPC could even fight against endotoxemia by increasing the level of SOD (Liang et al., 2008b). SDS is an oral solution that originated from modern empirical prescriptions (Zhang et al., 2010). Its primary effects are clearing heat and invigorating the spleen, and it is primarily used for dizziness, nausea, vomiting and other gastrointestinal reactions caused by HS. It has been proven to be effective on accelerating the cooling rate of HS patients and refreshing the brain (Shen et al., 1998).

Next, three types of CPM pills are introduced. Although traditional TCM pills have been made since ancient times, the present pills are manufactured using modern pharmaceutical processes and contain more stable compositions. Therefore, these modern TCM pills could be classified as Chinese patent medicines rather than herbal prescriptions or formulas. AGNHP, first recorded in the book of *Wen Bing Tiao Bian* (*Treatise on differentiation and treatment of seasonal warm diseases*), is a famous CPM pill for the treatment of various acute cerebrovascular diseases, such as encephalitis, meningitis, cerebral infarction, and intracerebral hemorrhage with the effects of removing heat and eliminating phlegm and toxic material (Zhang et al., 2022a). According to clinical research, AGNHP could lower the levels of serum TNF-α, IL-1, IL-6 and the APACHE II scores of HS patients and elevate the expression of heat shock protein 70 (Shen et al., 2020). Another study found that AGNHP could shorten the time of temperature reduction to 38.5°C and improve the rate of recovery of consciousness at 36 h (Shi et al., 2021b). LHRDP is a novel CPM pill based on XJS developed at the beginning of the 20th century in China. This pill could clear heat, awaken the brain, and invigorate the spleen (Wang et al., 2014b; Wang et al., 2014c). Therefore, it has been widely used to counter dizziness and gastrointestinal reactions caused by damp and heat and is a recommended medicine for travel. A study found that LHRDP could lower the high temperature, alleviate the hemoconcentration and decrease the mortality of HS rats with significant difference (Li et al., 2009). WZYZP, a pill with the effects of tonifying the kidney and securing the essence, was developed during the Tang Dynasty. It has been long used for the treatment of male infertility. Interestingly, a study found that WZYZP could strengthen the blood-testis barrier by upregulating the expression of ZO-1 and occluding via Akt signaling, A study found that WZYZP could alleviate spermatogenesis disorder induced by heat stress dependent on the Akt and NF-κB signaling pathways. Additionally, researchers have considered that the potential mechanism by which WZYZP enhances the heat stress resistance of Sertoli cells may entail the preservation of tight junctions (Hu et al., 2021b), the suppression of apoptosis, and the facilitation of cell differentiation and maturation (Xu et al., 2021).

## 5 Single Chinese herbs and associated extracts or monomers against HS

An herbal formula is made up of a variety of herbs according to the theory of compatibility. Due to the complex components of each herbal formula, identifying the single herb and associated extracts or monomers could elicit the key factors that antagonize HS and is beneficial to detect the specific mechanisms. According to a literature review, twenty-four types of single Chinese herbs and their associated extracts or monomers against HS were directly reported to be effective on HS (Table 3). It is worthy to note that many herbs are also part of the herbal formulas. These herbs with high concentration will be further analyzed and discussed. The subsequent content presents a categorical overview and analysis of twenty-four pharmaceutical substances of these herbs.

**TABLE 3:** Summary of representative single Chinese herbs and associated extracts or monomers for HS.

Single Chinese herbs		Main active components	References
Beidougen	*Menispermum dauricum DC.*	Total alkaloid	Xu et al. (1999)
Cangzhu	*Atractylodis Rhizoma*	Rhizoma Atractylodes macrocephala polysaccharide	Song et al. (2014), Luo et al. (2022)
Chuanxinlian	*Atractylodes lancea (Thunb.) DC.*	Andrographolide	Tan et al. (2007)
Danggui	*Angelica sinensis (Oliv.) Diels*	Ferulic acid	Cheng et al. (2018), He et al. (2019)
Fuzi	*Aconitum carmichaelii Debeaux*	Aconitum alkaloids	Huh et al. (2021)
Hongjingtian	*Rhodiola rosea L.*	Salidroside	Xiao and Li (2022)
Houpu	*Houpoea officinalis (Rehder & E.H.Wilson) N.H.Xia & C.Y.Wu*	Polyphenolic neolignans, magnolol, honokiol	Li et al. (2020a)
Huangbo	*Phellodendron amurense Rupr.*	Berberine	Jiang et al. (2017)
Huanglian	*Coptis chinensis Franch.*	Berberine	Moon et al. (2017)
Huangqi	*Astragalus membranaceus Fisch. ex Bunge*	Triterpene saponins, flavonoids, and polysaccharides	Shi et al. (2021a)
Huangqin	*Scutellaria baicalensis Georgi*	Baicalin	Guo et al. (2015)
Huoxiang	*Agastache rugosa (Fisch. & C.A.Mey.) Kuntze*	Ursolic acid	Luan et al. (2011)
Huzhang	*Reynoutria japonica Houtt.*	Resveratrol	Cheng et al. (2019a)
Jianghuang	*Curcuma longa L.*	Curcumin	Sahin et al. (2012)
Jiegeng	*Platycodon grandiflorus (Jacq.) A.DC.*	Saponins	Leng et al. (2019)
Lizi	*Castanea sativa Mill.*	Tannins	Liu et al. (2018b)
Luotuoci	*Alhagi camelorum Fisch.*	Phenolics, flavonoids, alkaloids, polysaccharides	Wang et al. (2019b)
Mahuang	*Ephedra Tourn. ex L.*	Ephedrine, pseudoephedrine	Kim et al. (2020)
Qinghao	*Artemisia carvifolia Besser*	Artesunate	Jiang and Zhang (2018)
Renshen	*Panax ginseng C.A.Mey.*	Ginsenosides	Hwang et al. (2015), Chei et al. (2020), Sandner et al. (2020)
Ruanzicao	*Arnebia euchroma (Royle ex Benth.) I.M.Johnst.*	Shikonin, alkannin	ZHANG (2020)
Sangye	*Morus cathayana Hemsl.*	4-O-Caffeoylquinic acid	Ganzon et al. (2018)
Shichangpu	*Acorus gramineus Aiton*	Asarone.	Li et al. (2020b)
Tengcha	*Ampelopsis grossedentata (Hand.-Mazz.) W.T.Wang*	Dihydromyricetin	Feng et al. (2019), Wang et al. (2021)

Beidougen (*Menispermum dauricum*) is a perennial, sinuous vine belonging to the Menispermaceae family with their primary distribution in China, Japan, North Korea, and Russia (Chen et al., 2020; Lv et al., 2020). It has been widely used in inflammatory diseases, cardiovascular, and thrombosis disorders. A study demonstrated that the total alkaloid of Beidougen could inhibit the increase in serum corticosterone of HS mice (Xu et al., 1999). Cangzhu (*Atractylodis Rhizoma*) is a traditional Chinese herb with immune regulating functions (Song et al., 2014; Luo et al., 2022). It could strengthen the spleen and Qi, eliminate dampness, calm the fetus, and promote diuresis. According to veterinary research, Cangzhu possessed the ability of anti-heat stress in spleen and intestine (Xu et al., 2017). In addition, the polysaccharide of Cangzhu was shown to have the protective effects on endoplasmic reticulum stress and apoptosis in HS-induced spleen injury (Zhu et al., 2011). Chanxinlian (*Andrographis paniculata*) is a type of herb with a strong bitter taste used to treat sore throats, common cold, and noninfective diarrhea (Dai et al., 2019). The primary component, andrographolide, has been shown to protect against HS with the effects of increasing the cooling rate, reducing mortality, and significantly decreasing the LPS concentration of HS mice blood. Additionally, its protective effects on HS were performed by regulating the expression of TLR4 and NF-κB and inhibiting apoptosis in HS mice. Another study also found that neo- andrographolide had a better effect against HS, especially at the dose of 5 mg/kg (Tan et al., 2007). In addition, its mechanism might derive from the upregulation effects of heat shock protein 70. Danggui (*Angelica sinensis*) is widely used to replenish blood, invigorate blood, stop pain, and moisten the intestine and is often called female ginseng (He et al., 2019). A Study on pharmacological effects of Dangui in Mice found that the extracts of Danggui had anti-fatigue, anti-anoxia and anti-high temperature effects on mice by increasing the living time (Huang et al., 2006). The primary active component of Danggui, ferulic acid, has been found to safeguard against intestinal epithelial barrier dysfunction induced by heat stress in IEC-6 cells through the Pl3K/Akt-mediated Nrf2/HO-1 signaling pathway (Cheng et al., 2018; He et al., 2019). Fuzi (*Aconitum carmichaelii*) is the active ingredient of the Geongangbujia-Tang decoction and has been found to exert inhibitory effects on heat stress-induced inflammation in mice (Huh et al., 2021). Hongjingtian (*Rhodiola rosea*) is intended to rectify insufficient Qi, fortify pulmonary function, impede hemorrhaging, alleviate blood stagnation, and mitigate inflammation in TCM(Pu et al., 2020). Salidroside the primary active component of Hongjingtian, is a naturally occurring phenylpropanoid glycoside widely used as an anti-hypoxia, anti-fatigue, anticancer activity, anti-inflammation, and anti-oxidation agent. A new study indicated that salidroside could reduce the degree of cardiac impairment by up-regulating the peroxisome proliferator-activated receptor-γ coactivator-1α and manganese-SOD (Xiao and Li, 2022). Another study found that salidroside could protect the liver of HS mice by inhibiting the production of HMGB1 in Kupffer cells (Zhang et al., 2018). It has also been shown to help increase the expression of heat shock protein 70 and reduce the volume of cerebral infraction. Houpu (*Houpoea officinalis*) and Shichangpu (*Acorus gramineus*) have also been shown to have protective effects on blood pressure in HS rat by nose inhalation (Li et al., 2020b). Berberine, the important component of Huangbo (*Phellodendron amurense*), was proved to be effective on suppressing the expression of heat shock protein 70 and TNF-α and decreasing body temperature of HS(Jiang et al., 2017). Dietary supplementation with beberine could enhance immunity and reduce oxidative stress (Zhang et al., 2013). Huanglian (*Coptis chinensis*) is a traditional herb with potent antipyretic activities widely used in many herbal formulas to lower fever, soothe coughs, relax blood vessels, and prevent mycoses. Treatment with Huanglian could reduce the heat stress-induced expression of NF-κB, TNF-α and interleukin-1β in the hippocampus (Moon et al., 2017). The study demonstrated that Huanglian could protect the brain against HS-mediated damage via amelioration of hyperthermia and neuroinflammation in mice. In addition, the compatibility of Huanglian and Zhuyu (*Evodia rutaecarpa*) could significantly enhance heat tolerance and mitigate HS-induced injury and death (Gao et al., 2010). Interestingly, berberine is also the primary active component of Huanglian with effective anti-heat toxicity. Huangqi (*Astragalus membranaceus*) is one of the most widely used fundamental herbs in TCM with immunomodulation, anti-inflammation, antioxidation, tonic action, hepatoprotection, diuresis, anti-diabetes, anticancer, anti-photoaging, and expectorant actions. Its extract of astragalus polysaccharides could increase heat stress tolerance and related gene expression (Abarike et al., 2020; Zeng et al., 2020). It could also improve the expression of heat shock protein 70 and HIF-1α mRNA to heat stress (Shi et al., 2021a). Baicalin, the primary component of Huangqin (*Scutellaria baicalensis*), has been reported to protect mouse testis from HS-induced injury by increasing anti-oxidative enzyme activities and blocking the Fas/FasL pathway (Guo et al., 2015). It could also protect Sertoli cells from HS-induced apoptosis via activation the Fas/FasL pathway and heat shock protein 72 expression (Sui et al., 2019). Huoxiang (*Agastache rugosa*) has been proven to prevent heat stress induced-apoptosis in a rat intestinal epithelial cell line (IEC-6) (Luan et al., 2011). In addition, it could also help recover splenic lymphocytes from the immunosuppression induced by HS. Huoxiang’s priamry active ingredient, ursolic acid, mitigates heat stress-induced lung injury and cardiac injury by regulating endoplasmic reticulum stress signaling (Yang et al., 2014b). Resveratrol, the primary active component of Huzhang (*Reynoutria japonica*), has been proven to be protective in numerous studies (Cheng et al., 2019a; Cheng et al., 2019b; Khafaga et al., 2019). By enhancing oxidative status and mitigating inflammation in heat-stressed rats, it has the potential to ameliorate intestinal injury and dysfunctions. The administration of dietary resveratrol has been observed to impede the heightened activation of innate immunity and inflammatory response in the spleen of yellow-feather broilers subjected to heat stress. A rat model showed that resveratrol inhibits hepatic damage induced by heat stress by regulating inflammation and oxidative stress. In addition, it has been widely used in agriculture to protect animals against heat stress. Curcumin is a polyphenol compound extracted from the rhizomes of Jianghuang (*Curcuma longa*) and has been widely used to treat diseases. The administration of curcumin supplements could ameliorate cardiac injury induced by heat stress in mice (Szymanski et al., 2018). In addition, its mechanism could perform via inhibition of oxidative stress and modulation of the Nrf2/HO-1 pathway (Sahin et al., 2012). According to another study, the administration of curcumin exhibited a dose-dependent amelioration of spermatogenic disorders that are induced by scrotal heat stress in mice (Yon et al., 2016). The saponins from Jiegeng (*Platycodon grandiflorus*) have various beneficial activities that include anti-inflammatory, anti-cancer, and anti-oxidant properties. The study demonstrated that the saponins found in Jiegeng could mitigate the negative effects of scrotal heat on spermatogenesis in mice by inhibiting oxidative stress and apoptosis (Leng et al., 2019). Tannins, which are polyphenolic compounds that are soluble in water, are derived from Lizi (*Castanea sativa*) and have been documented to exhibit antioxidant, antimicrobial, and anti-inflammatory properties. According to a study, the regulation of the intestinal morphology, barrier function, pro-inflammatory cytokine expression, microflora, and antioxidant capacity could potentially mitigate the adverse impacts of heat stress on growth performance and intestinal function of broilers (Liu et al., 2016; Liu et al., 2018b). Luotuoci (Alhagi sparsifolia) extracts possess a protective effect on liver injury of HS rats. It could prolong the survival time and alleviate liver injury by inhibiting NF-κB, Xaspase-3 expression and enhancing heat shock protein 70 expression (Wang et al., 2019a). Mahuang (*Ephedra*) has been shown to inhibit heat-induced proinflammatory factors and promote hypothalamic homeostasis (Kim et al., 2020). Artesunate is a derivative of Qinghao (*Artemisia carvifolia*) and could attenuate heat stress-induced injury via the activation of ERK/P53 signaling pathway and suppression of the expression levels of Bax and MDM2 (Jiang and Zhang, 2018). A previous study also found that it could decrease the increasing rate of the anus temperature, reduce the moral rate, and significantly depress the content of LPS in mice blood by down-regulating the expression of NF-κB mRNA and up-regulating the expression of heat shock protein 70 (Zhang et al., 2006). Renshen (*Panax ginseng*) is a highly regarded herbal medicine in TCM, and its extracts are rich in ginsenosides that have multiple functions. Many studies have demonstrated that Renshen and its ginsenosides extracts could significantly ameliorate HS-induced injury (Hwang et al., 2015; Chei et al., 2020; Sandner et al., 2020). Renshen could directly decrease the malondialdehyde contents and ROS associated genes of HS rats and then inhibit the abnormal conditions of HS. Its extracts could prevent hepatic oxidative stress and inflammation induced by HS(Kim et al., 2015). Ginsenosides could also improve intestinal barrier integrity by supporting heat shock response *in vitro*. In addition, various formulas with anti-heat stress effects contain Renshen, which have been mentioned above. Ruanzicao (*Arnebia euchroma*) is widely used to treat inflammatory diseases. A network pharmacology study found that Ruanzicao has the potential to impede inflammatory responses, enhance blood circulation, and safeguard the central nervous system, thereby ameliorating damage to multiple organs, and serving as a protective agent against HS(Ou et al., 2017; Zhang, 2020). Sangye (*Morus cathayana*) is widely used as an herbal tea to prevent HS. 4-O-Caffeoylquinic acid as an antioxidant marker that could be used to protect HS(Ganzon et al., 2018). Dihydromyricetin is a flavonoid that is also known as ampelopsin from Tengcha (*Ampelopsis grossedentata*). It was considered to be protective against hyperthermia-induced apoptosis in human myelomonocytic lymphoma cells via MAPKs and PI3K/AKT signaling pathway (Wang et al., 2021). It has also been reported to be able to attenuate heat stress-induced apoptosis in epithelial cells by suppressing mitochondrial dysfunction (Feng et al., 2019).

## 6 External therapies against HS

According to various studies, external therapies, such as Gua sha, Tui na and acupuncture, have been found to purge internal heat and cool or refresh body with unexpected effects on some HS patients. Gua Sha and Tui na can promote blood circulation, expand capillaries, increase the secretion of sweat glands, and achieve the role of reducing hyperthermia and dredging meridians. The mechanism of acupuncture for HS can be summarized in the following two aspects. Acupuncture can improve the dysfunction of the body temperature regulation center and speed up body heat dissipation. Additionally, acupuncture can improve blood distribution throughout the body Acupuncture can enhance blood circulation in the skin, increase heat dissipation, and regulate blood distribution in the visceral organs. Research has shown that Gua sha combined blood-letting after HS can improve survival by reducing systemic inflammation, hypercoagulable states, tissue ischemia, and multiple organ damage (Aprile et al., 2015; Tu et al., 2015). XNJI combined with acupuncture on HS could help reduce the recovery time of consciousness and alleviate the injury of organs. Spermatogenic cells were found to be increased due to acupuncture in rats suffering from HS(Purnama et al., 2018; Upadhyay et al., 2019). In addition, acupuncture has been proven to be particularly beneficial for heat cramp. Moreover, a study found that cold-water dousing with ice massage could accelerate the cooling rate and improve survival of HS(Mcdermott et al., 2009).

## 7 Discussion

**FIGURE 1:**
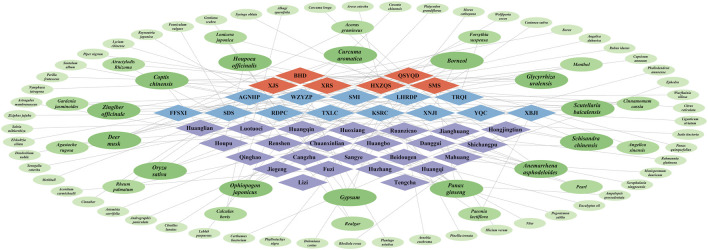
The relationship of herbal formulas, CPMs and single Chinese herbs. The red diamonds represent the different herbal formulas. The blue diamonds represent the different CPMs. The purple diamonds represent the different Chinese herbs. The first circle dark green circles represent the Chinese herbs mentioned in herbal formulas, CPMs and single Chinese herbs more than three times. The second green circles represent the Chinese herbs mentioned in herbal formulas, CPMs and single Chinese herbs two times. The third light green circles represent the Chinese herbs mentioned in herbal formulas, CPMs and single Chinese herbs one time.

A total of six herbal formulas, 13 CPMs and 24 Chinese herbs have been found to be effective to protect and prevent HS. Moreover, some external therapies are also considered to be useful in the treatment of HS. According to Cytoscape network analysis (Figure 1), we found that some Chinses herbs were mentioned in herbal formulas, CPMs, single Chinese herbs and associated studies. A total of 14 Chinses herbs were mentioned more than three times. In addition, 14 Chinese herbs were mentioned two times. These repeated Chinese herbs are quite significant as they are ingredients of various herbal medicines. *Borneol, P. ginseng, Glycyrrhiza uralensis, S. baicalensis, Anemarrhena asphodeloides, Ophiopogon japonicus, Deer musk, C. chinensis, Curcuma aromatica, Schisandra chinensis, Gypsum, Oryza sativa* and *Zingiber officinale* are mentioned more than three times and tend to be the components of various herbal formulas and CPMs. *Borneol* is in the composition of XJS, FFSXI, XNJI TXLC, AGNHP, and LHRDP. *Panax ginseng* is the composition of SMS, SMI, KSRC, and TXLC. *Glycyrrhiza uralensis* is in the composition of HXZQS, BHD, QSYQD, and LHRDP. *Scutellaria baicalensis* is in the composition of TRQI, YQC, and AGNHP. *Anemarrhena asphodeloides* is in the composition of BHD, QSYQD, KSRC, and RDPC. *Ophiopogon japonicus* is in the composition of SMS, QSYQD, SMI, and RDPC. *Deer musk* is the composition of is in the composition of XJS, FFSXI, XNJI, and AGNHP. *Coptis chinensis* is in the composition of QSYQD, YQC, and AGNHP. *Curcuma aromatica* is in the composition of FFSXI, XNJI, and AGNHP. *Schisandra chinensis* is in the composition of SMS, SMI, and WZYZP. *Gypsum* is in the composition of BHD, KSRC, and RDPC. *Oryza sativa* in is the composition of BHD, QSYQD, and LHRDP. *Zingiber officinale* is in the composition of XJS, SDS, and LHRDP. These Chinses herbs with high occurrence could be considered the core herbs against HS. Among these herbs, *P. ginseng, S. baicalensis, C. chinensis and C. aromatica* have been investigated to be effective against HS based on single herb experiments. These four Chinese herbs seem to be worthy of vigorous study. It is worthy to note that most of these have the ability of clearing heat and detoxifying. The rest of the herbs could reinforce Qi and promote the production of body fluids. This also echoes the pathophysiological mechanism of heat stroke. Clearing heat could attenuate the injury induced by heat stress. Strengthening Qi and producing body fluids could protect organs and provide life sustainment. In fact, patients with HS do require relief of thermal damage and multiorgan support at a later stage in the ICU. Traditional Chinese medicine and modern Western medicine have interesting resonances here. And it suggests that it is of great significance for us to find a solution to HS from a traditional Chinese medicine perspective. In addition, we should pay special attention to the application of CPMs. Currently, even in the home country of TCM, there remains some controversy regarding CPMs, and it is regarded as a double-edged sword. They play an important role in preserving and promoting traditional knowledge and practices of Chinese herbal medicine, helping to maintain the continuity of ancient medical traditions, and provides access to traditional remedies for various ailments. However, some CPMs have been found to contain heavy metals, toxins, or undeclared pharmaceutical ingredients. These safety concerns highlight the importance of quality control and regulation in the production and distribution of CPMs. While many CPMs have been used for centuries and are believed to be effective by practitioners and patients, scientific evidence supporting their efficacy is limited. The lack of rigorous clinical trials and standardized research methodologies poses challenges in evaluating their effectiveness. This is especially true for injections of CPMs that have been a topic of debate and scrutiny. Concerns have been raised about quality control, potential contaminants, and the standardization of manufacturing processes. Additionally, scientific evidence supporting their effectiveness is limited compared to conventional Western medicine. It is important to note that the double-edged effects of Chinese patent medicine vary depending on factors such as product quality, proper usage, individual response, and adherence to regulatory standards. Among the CPMs listed above, SMI, XBJI, and YQC have clinical trial evidence and can be recommended for safe use. Other CPMs have clinical experience reports and animal experiments as the basis. We believe that these drugs deserve to be validated in rigorous clinical trials in the future. Since various herbal formulas, CPMs, and Chinese herbs have therapeutic potential against HS, high-throughput screening should be performed to determine the effective medicines. Some TCM medicines that have been shown to be effective should be studied to find the specific mechanisms.

## 8 Conclusion

In summary, as shown in Figure 1, various herbal formulas, CPMs and Chinese herbs have therapeutic potential against HS. These TCM medicines could cleat heat, resolve damp, reinforce Qi, nourish Yin, and promote the production of body fluids. In addition, research based on modern experiments have also shown that these medicines could alleviate HS-induced injury, improve mortality, and suppress oxidative stress and inflammation associated pathways. As we mentioned at the beginning, few pharmacologic agents have been demonstrated to be able to treat HS, and TCM has a long history and clinical practice on HS. Therefore, these TCM medicines and external therapies should be further studied to provide alternative complementary treatment options for HS patients. Along with this further exploration, it is hoped that a thorough understanding of the application of TCM on HS treatment strategies and its ability to target various aspects of pathogenesis of HS will reveal the profound therapeutic benefits of TCM.
